# Chrysin Modulates Behavior and Hippocampal Histopathology in Adult Male Hypoandrogenic Rats: The Regulatory Role of miR‐30a, miR‐375, and miR‐204

**DOI:** 10.1002/brb3.70529

**Published:** 2025-05-07

**Authors:** Amir‐Hossein Ebadi, Maryam Moghimian, Seyd‐Hossein Abtahi‐Evari, Zahra Saadatian, Alireza Fathi, Malihe Soltani

**Affiliations:** ^1^ Student Research Committee Gonabad University of Medical Sciences Gonabad Iran; ^2^ Department of Physiology, Faculty of Medicine Gonabad University of Medical Sciences Gonabad Iran; ^3^ Department of Biochemistry and Nutrition, Faculty of Medicine Gonabad University of Medical Sciences Gonabad Iran; ^4^ Department of Anatomy, Faculty of Medicine Gonabad University of Medical Sciences Gonabad Iran

**Keywords:** behavior, chrysin, hippocampal, hypoandrogenic, microRNA

## Abstract

**Objective:**

Hypoandrogenism, a condition linked to oxidative stress and hippocampal cell death, can lead to learning and memory disorders in aging men or those with hypogonadism. Recently, microRNAs such as miR‐30a, miR‐375, and miR‐204 have been identified as regulators of cell death and memory formation. Flavonoid antioxidants that modulate microRNA expression offer a potential therapeutic approach for neurodegenerative conditions. This study examines the effects of the flavonoid chrysin on these microRNAs, cell death processes, and memory under hypoandrogenic stress.

**Methods:**

Rats were assigned to six groups: control, hypoandrogenic, hypoandrogenic treated with chrysin (50 mg/kg and 75 mg/kg), and chrysin treated alone (50 mg/kg and 75 mg/kg). After 14 days, memory changes were assessed using the Morris water maze test, along with evaluations of oxidative stress enzymes, apoptosis and autophagy, miR‐30a, miR‐375, and miR‐204 gene expression, and the number of dark neurons.

**Results:**

Hypoandrogenism increased apoptosis and autophagy gene expression, dark neurons, oxidative stress enzymes, and decreased memory and microRNA expression compared to controls. Treatment with chrysin (75 mg/kg) significantly reduced apoptosis and autophagy gene expression, dark neurons, and oxidative stress enzymes, and improved memory and learning. This group also showed modulation of miR‐30a, miR‐375, and miR‐204 levels compared to the hypoandrogenic group.

**Conclusion:**

Hypoandrogenism led to neurodegeneration and memory impairment, likely due to oxidative stress affecting miR‐30a, miR‐375, and miR‐204 regulation. Chrysin (75 mg/kg) potentially mitigates cell death processes and memory deficits in hypoandrogenism by modulating these microRNAs.

## Graphical abstract

Hypoandrogenism with expression disorder of miR‐30a, miR‐375, and miR‐204 leads to neurodegeneration, apoptosis, and autophagy, as well as learning and memory disorders. Chrysin mitigates cell death processes and memory deficits in hypoandrogenism by modulating these microRNAs.

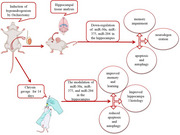

## 1 Introduction

In recent decades, a significant body of research has reported on the effects of hypogonadism in regions of the brain that possess androgen receptors, including the hippocampus (Kight and McCarthy 2020). Androgens play a crucial role in memory formation and learning by influencing neuronal proliferation and survival, regulating dendritic spine density, and synaptogenesis in the CA3 region and dentate gyrus of the hippocampus (Atwi et al. 2016). It has been established that androgens, including testosterone, contribute to stabilizing the antioxidant system and inhibiting apoptosis by controlling the production of reactive oxygen species (ROS) and maintaining mitochondrial respiratory chain function in hippocampal neurons (Fanaei et al. 2014; Muthu and Seppan 2020). In the hippocampus, cell death can be induced not only through apoptosis but also via excessive autophagy. Autophagy is a catabolic process that aids in neuronal homeostasis and synaptic plasticity in hippocampal neurons (Li et al. 2014). During this process, autophagosomes—membrane‐bound vesicles involved in autophagy—are formed through the expression of autophagy‐related genes (ATG), including LC3II, Beclin‐1, Atg5, Atg7, and Atg12 (Mizushima 2007). Disruptions in the expression of autophagy genes have been observed in numerous neurodegenerative diseases, as well as in memory and learning disorders (Nixon 2013). However, the precise relationship between memory impairment and the autophagy process during hypogonadism remains unclear.

To address this, the present study evaluated the expression of several common microRNAs (miRNAs) involved in regulating both the autophagy process and memory, specifically miR‐30a (Xu, Wang, Tan, and Jing 2012), miR‐375, and miR‐204 (Frankel and Lund 2012). miRNAs are a class of small non‐coding regulatory RNAs, approximately 22 nucleotides in length, that mediate gene silencing post‐transcriptionally by targeting specific RNA sequences. They also play roles in regulating growth, proliferation, differentiation, and cell death (Su et al. 2015). The aforementioned miRNAs are specifically expressed in the hippocampus and play significant roles in modulating neural responses, memory formation, and post‐transcriptional regulation of various autophagy‐related genes (X. Liu et al. 2015; Shan et al. 2017; Tao et al. 2021). Altered expression of these miRNAs has been shown to underlie many neurodegenerative diseases (Wang et al. 2012).

Numerous therapeutic targets based on antioxidants aimed at modulating miRNA expression during neurodegenerative diseases have been reported (Nelson, Wang, and Rajeev 2008). Among these, flavonoid compounds have garnered attention for their potential to prevent and treat memory disorders, modulate autophagy, and regulate various miRNA expressions (Singh, Raza, Parveen, Meena, and Luqman 2021). Chrysin (5.7‐dihydroxyflavone) is one of the most active natural flavonoids known for its ability to protect against cognitive impairments, neurogenesis, and memory issues (Ahmed et al. 2018). Due to its antioxidant, anti‐inflammatory, and anti‐apoptotic properties, this flavonoid has emerged as a suitable candidate for studies related to neurological disorders such as epilepsy, memory deficits, motor behavior disorders, cerebral ischemia, Parkinson's disease, and Alzheimer's disease (Angelopoulou, Pyrgelis, and Piperi 2020; C. Liu et al. 2021; Nabavi et al. 2015; Yao et al. 2014). Some studies have tested chrysin's potential to combat oxidative stress and restore neurogenesis in hippocampal disorders (Borges Filho et al. 2016). Furthermore, the role of this flavonoid in modulating the expression of certain miRNAs involved in proliferation, differentiation, metastasis, autophagy, and apoptosis has been well documented (Mohammadian et al. 2017). However, the extent to which chrysin affects memory and neurogenesis in hypogonadal rats and whether the underlying molecular mechanisms are related to the regulatory miRNAs miR‐30a, miR‐375, and miR‐204 remain unknown. Thus, the present study aims to evaluate the effects of chrysin on memory and hippocampal histopathology through the regulation of miR‐30a, miR‐375, and miR‐204 in hypogonadal rats.

## 2 Material and Methods

This experimental study was carried out using 48 adult male Wistar rats, each weighing between 250 and 300 grams. The rats were housed in the animal facility at Gonabad University of Medical Sciences, where they were kept under standard conditions: a 12‐h light/dark cycle, a stable temperature of 22 ± 2 degrees Celsius, and adequate ventilation. Throughout the housing and experimental phases, the rats had unrestricted access to food and water. All research activities adhered to ethical standards for the care and use of laboratory animals, following the guidelines established.

### 2.1 Experimental Groups

In the present study, the rats were randomly divided into six groups of eight based on hypogonadal treatment. The grouping is as follows:

Group 1: Sham group, where only the scrotal wall was incised and sutured without inducing hypogonadism. Additionally, gavage with corn oil was performed (Sham).

Group 2: Induction of hypogonadism through bilateral orchiectomy surgery accompanied by gavage with corn oil.

Group 3: Induction of hypogonadism + treatment with chrysin dissolved in corn oil (50 mg/kg/day) administered via gavage for 14 days.

Group 4: Induction of hypogonadism + treatment with chrysin dissolved in corn oil (75 mg/kg/day) administered via gavage for 14 days.

Group 5: Treatment only with chrysin dissolved in corn oil (50 mg/kg/day) administered via gavage for 14 days.

Group 6: Treatment only with chrysin dissolved in corn oil (75 mg/kg/day) administered via gavage for 14 days.

### 2.2 Surgical Procedure of Orchiectomy

To induce hypogonadism, the animals were anesthetized via intraperitoneal injection of urethane (1.2–1.5 mg/kg), and a longitudinal incision was made on both sides of the scrotal wall. After retracting the layers of the scrotum, the testes were separated from the surrounding fascia. The testes were then excised by cutting the remnants of the gubernaculum from the tunica vaginalis. To prevent infection during the surgical procedure, antiseptic materials were used, and each rat received an injection of 0.2 mL of penicillin (6.3.3). Analgesics such as carprofen (5 mg/kg) and buprenorphine (0.02 mg/kg) were also administered to alleviate pain (Pronina et al. 2020). Following this, treatment with chrysin was conducted at two doses of 50 and 75 mg (Oriquat et al. 2023) daily for 14 days. In this study, chrysin was dissolved in corn oil and administered to the rats via gavage. At the end of the treatment period (day 14) (Shoieb et al. 2018), a memory assessment was performed using the morris water maze (MWM) test.

### 2.3 Memory Assessment Method via Morris Water Maze (MWM)

The MWM consists of a water tank with a diameter of 180 cm and a depth of 60 cm, filled to approximately half with water at a temperature of 22°C. The tank is hypothetically divided into four equal quadrants. A black escape platform is positioned 1 cm below the water surface in the center of the northern quadrant. Animals were placed in one of four randomly selected positions in the water, and the time taken to escape onto the platform (escape latency) was measured. The rats underwent regular training for four consecutive days. After locating the platform, the rats were allowed to remain on it for 30 s. If a rat failed to find the platform within 60 s, it was guided to it by the researcher. The maze was set in a room containing various spatial cues that remained constant throughout the trials and were visible to the animals in the maze. This setup was monitored using a tracking camera positioned 180 cm above the center of the water maze to record escape latency, swimming distance (distance traveled), and time spent in the target quadrant. On day five, each rat underwent a 60 s probe trial and a visible platform trial. During the probe trial, no platform was present, while in the visible platform trials, the platform was covered with aluminum foil. In each trial, parameters such as escape latency, distance traveled, time to reach the target quadrant, and average swimming speed were recorded (Othman et al. 2022).

### 2.4 Biochemical Parameters

#### 2.4.1 MDA (malondialdehyde)

After the experimental period, 0.20 mL of collected serum was transferred to a microtube containing 3.0 cm^3^ of glacial acetic acid. Then, thiobarbituric acid (TBA) was added to the microtube containing sodium hydroxide (NaOH) at a concentration of 2%. The TBA added was at a concentration of 1%. The microtube was immersed in boiling water for 15 min. After cooling, a spectrophotometer was used to measure the absorbance of the pink color in both test and standard solutions at a wavelength of 532 nm (Lapenna et al. 2001).

#### 2.4.2 SOD (superoxide dismutase) and GPX (glutathione peroxidase)

The levels of SOD and GPX in serum were measured using an ELISA Reader (Model: ABER‐2, China)—AccuBioTech. Measurements were conducted according to protocols provided by the manufacturers (Randox and Ransod, UK). The evaluation of GPX levels utilized the Paglia‐Valentine technique, which involves the oxidation catalysis of glutathione (GSH) with cumene hydroperoxide. Measurement of SOD levels involved using xanthine and xanthine oxidase (XOD) to produce superoxide radicals that subsequently interact with 2‐(4‐iodophenyl)‐3‐(4‐nitrophenol)‐5‐phenyl tetrazolium chloride (I.N.T), forming a red formazan color (Soltani et al. 2023).

### 2.5 Methodology for Real‐Time Analysis

After isolating the hippocampus from the brain, the hippocampal samples were homogenized using a Polytron PT 1200E homogenizer from Switzerland. Total RNA extraction from the hippocampal tissue was performed according to the manufacturer's instructions provided with the Super RNA Extraction Kit from Yekta Tajhiz, Iran. RNA purity was verified through agarose gel electrophoresis. The extracted RNA was then reverse‐transcribed into cDNA using the cDNA Synthesis Kit following the manufacturer's guidelines (Yekta Tajhiz, Iran). Real‐time PCR (RT‐PCR) (Reverse transcription polymerase chain reaction) was subsequently carried out on a 48‐well optical reaction plate using an Applied Biosystems StepOne system from Foster City, USA. The primary components of the real‐time PCR mixture included 1 µL of template cDNA, 0.2 µM of each forward and reverse primer, 3.6 µL of sterilized water, and 5 µL of SYBR Green from Ampliqon, Denmark. GAPDH was used as a housekeeping gene. To compare mRNA expression levels among the groups, the 2‐∆∆CT method was used, where ∆∆CT represents the differential expression of genes such as beclin‐1, caspase‐3, MAP LC3β, Atg5, Atg7, Atg12, and GAPDH. Gene amplification was conducted using an optimized protocol as follows: an initial denaturation at 95°C for 10 min, followed by 40 cycles of 15 s at 95°C, 30 s at 58°C, and 30 s at 72°C, concluding with 15 s at 95°C and a final step at 55°C for 1 h. The oligonucleotide primer sequences used for RT‐PCR were obtained from OligoGen, South Korea (Roshanaee et al. 2022).

### 2.6 Measurement of miRNA Expression by Stem Loop RT‐qPCR Method

The expression measurement of miR‐30a, miR‐375, and miR‐204 miRNAs was done using the stem‐loop RT‐qPCR method. In this method, cDNA synthesis is done using specific stem‐loop primers for each miRNA. These primers are designed to have a fixed sequence at their 5' end so that the universal reverse primer can be connected to this sequence in qPCR. Therefore, after cDNA synthesis, a common reverse primer and a specific miRNA forward primer are used to perform the qPCR reaction. Also, the RNU6 gene was used as a housekeeping gene to measure miRNA expression. The sequence of common stem‐loop, forward, and reverse primers is given in the table. Each cDNA synthesis reaction in a volume of 10 µL includes 0.5 µL of RT enzyme (Thermo Scientific, USA), 2 µL of RT enzyme buffer, 1 µL of stem‐loop primer, 1.5 µL of dNTP (Thermo Scientific, USA), 1 µL of RNA (about 500 ng), and 4 µL of nuclease‐free water. This reaction was carried out according to the following schedule: 30 min at 16°C, 30 min at 42°C, and 5 min at 82°C (Saadatian et al. 2023). The oligomer sequences of the forward and reverse primers used in RT‐PCR are as follows:

***Bax*** *(F primer: TTG CTA CAG GGT TTC ATC CA / R primer: GAG TAC CTG AAC CGG CAT CT)*,
***Bcl_2_*** *(F primer: GCTACCGTCGTGACTTCGC / R primer: CCCCACCGAACTCAAAGAAGG)*,
***Caspase3*** *(F primer: TGTGCTCCAGGCTTCCTTAATC / R primer: AGGCTTATGGGAAATGCTGGAC)*,
***Beclin‐1*** *(F primer: CGAAAGGTGGTGGCAGAAAAC/ R primer: ACTATATTCTCGCTGGTACTGAGC)*,
***Lc3 β*** *(F primer: TCAGTGAGAGCTGCCTCTGTC/ R primer: AGCAGTGGGGATTTACACAGTG)*,
***Atg5*** *(F primer: AGATCACAGTTCTGGGATGC/ R primer: TCAGGCGGTAGAGATCGTAG)*,
***Atg7*** *(F primer: TGTCTTGCAGCATCCTGAG/ R primer: TCAAGAACTTTGGATGAACAGG)*,
***Atg12*** *(F primer: GCACTCATCGACTTCATCAG/ R primer: ACTGCCAAAACACTCATATAGAG)*,
***GAPDH*** *(F primer GGTCTACATGTTCCAGTATGACTC/ R primer: CATTTGATGTTAGCGGGATCTCG)*,
***MIR‐375‐3P*** *(F primer: GTCGTATCCAGTGCAGGGTCCGAGGTATTCGCACTGGATACGACTCACGC/ R primer: CCCTTTGTTCGTTCGGCTC)*,
***MIR‐204‐5P*** *(F primer: GTCGTATCCAGTGCAGGGTCCGAGGTATTCGCACTGGATACGACGGCAT*
*/ R primer: CCCCTTCCCTTTGTCATCCT)*,
***MIR‐30A*** *(F primer: GTCGTATCCAGTGCAGGGTCCGAGGTATTCGCACTGGATACGACCTTCCA/ R primer: CCCCCCTGTAAACATCCTC)*,
***U6*** *(F primer: GTCGTATCCAGTGCAGGGTCCGAGGTATTCGCACTGGATACGACAAAAAT/ R primer: GCTTCGGCAGCACATATACTAAAAT)*,

### 2.7 Toluidine Blue Staining

At the end of the experimental period, the cerebral hemispheres were removed from the skull. The hemispheres were separated by sagittal cuts and fixed in a formalin solution for 72 h. Following fixation, a gradual process of ethanol, xylene, and liquid paraffin (Merck, Germany) infiltration was performed on the cerebral hemispheres. After paraffin embedding, brain tissue samples were sectioned to a thickness of 4 micrometers using a microtome (GMI‐Leitz1512, USA). The obtained slides were then subjected to deparaffinization and hydration. Subsequently, toluidine blue staining was utilized to identify dark neurons (DNs). DNs are a type of degenerative cell characterized by hyperbasophilia and hyper electron density in histological studies. These changes are observed in certain types of neural injuries. For toluidine blue staining, brain sections on slides were stained in a 0.03% toluidine blue solution (pH = 1.5) for 1–2 min after air drying, followed by washing with dH_2_O. They were then decolorized in 75% ethyl alcohol and dehydrated in 95% and 100% ethyl alcohol. Afterward, the sections were cleared in xylene for 2 to 3 min. Finally, a lamella was mounted on the slide, which was examined using a light microscope at a magnification of 400x (Asiaei et al. 2017).

### 2.8 Statistical Analysis

Statistical analysis was performed using SPSS 20 (IBM, USA). The Kolmogorov‐Smirnov test was utilized to determine the normal distribution of the data. All data were presented as mean ± standard deviation. One‐way analysis of variance (ANOVA) was employed to compare the values of DNs, systemic factors, and data from the MWM test, followed by Tukey's post hoc test. The independent t‐test was used for comparing and analyzing RT‐PCR technique data. A statistical significance level of p ≤ 0.05 was established.

## 3 Results

### 3.1 Number of DNs in the Hippocampus

**FIGURE 1:**
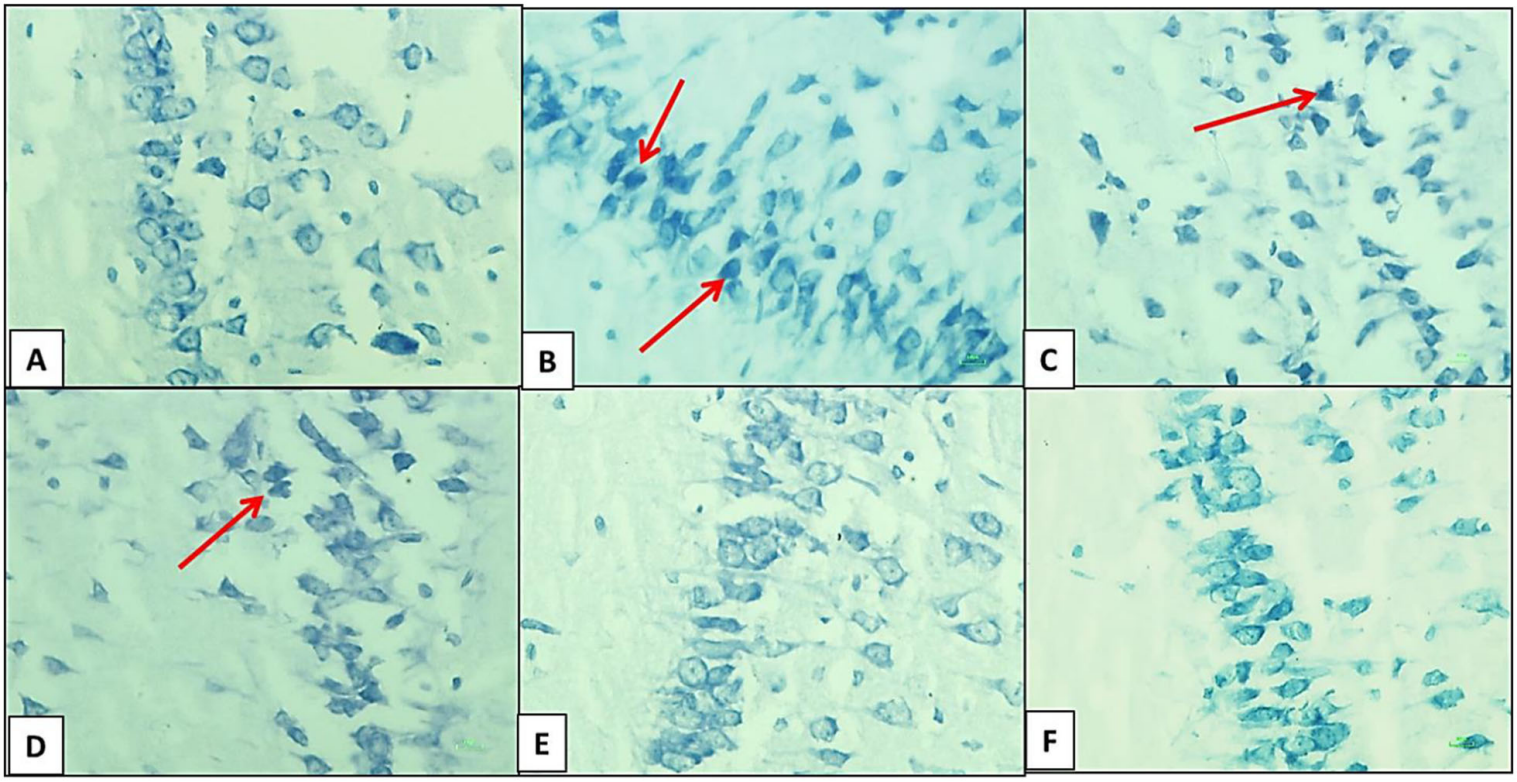
Histological findings of chrysin treatment following hypoandrogenic induction on all study groups' hippocampi using the toluidine blue staining method. A: control group, B: hypoandrogenic group, C: hypoandrogenic+chrysin50mg group, D: hypoandrogenic+chrysin75mg group, E: chrysin50mg group, and F: chrysin75mg group. The red arrows indicate the dark neurons in the hippocampus tissue.

**FIGURE 2:**
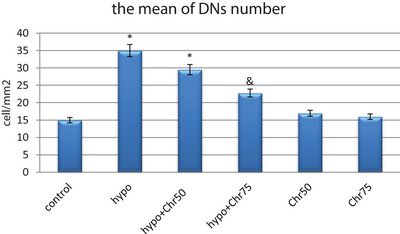
Evaluation of chrysin treatment following the hypoandrogenic induction on the hippocampus tissue by measuring the mean number of dark neurons (DNs) per unit area cell/mm2 in study groups. Values are mean ± SD. p < 0.05 are significant,* p < 0.05 versus control, and p < 0.05 versus hypoandrogenic group.

The number of DNs per unit area (cells/mm^2^) in the hippocampus and dentate gyrus was counted. The average number of DNs in the hippocampus across experimental groups was then compared. A small number of DNs were found in various regions of the hippocampus in the control group. In comparison with the control group, the average number of DNs per unit area in the hippocampus and dentate gyrus significantly increased in the hypoandrogenic group. Additionally, treatment with chrysin in the hypoandrogenic + chrysin group at a dose of 75 mg resulted in a significantly lower number of DNs in the aforementioned brain regions compared to the hypoandrogenic group (Figures 1 and 2) (p < 0.05).

### 3.2 Results of the MWM Test

#### 3.2.1 Escape Latency During Training and Test Days in MWM

**FIGURE 3:**
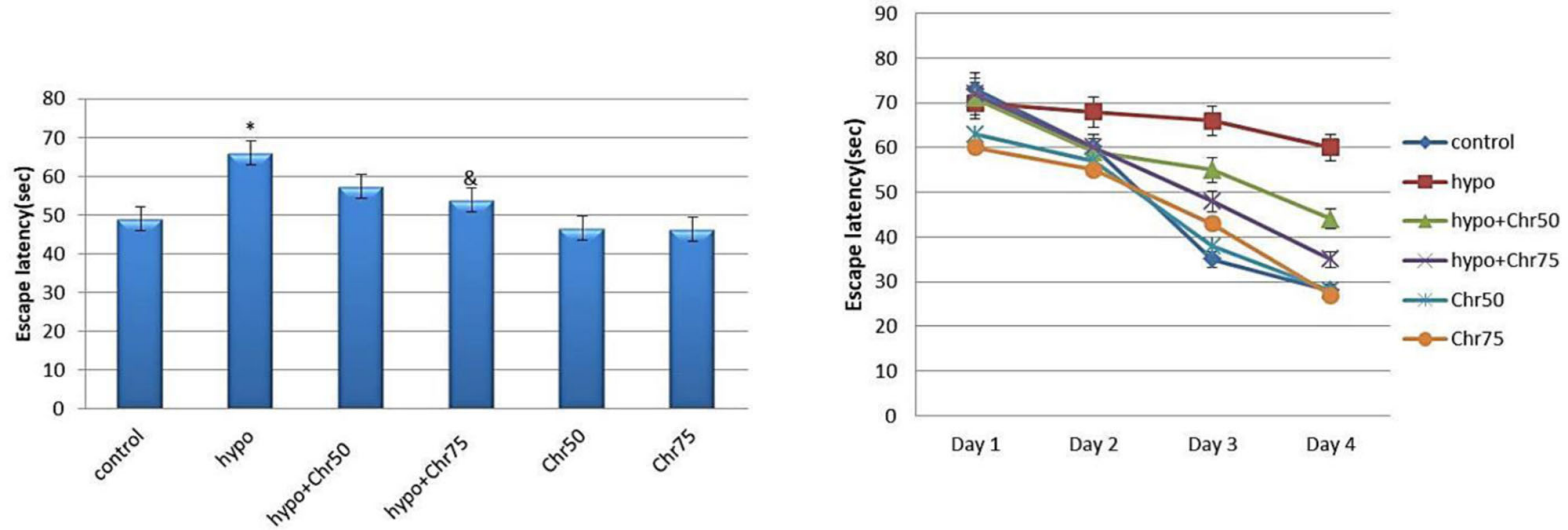
Evaluation of escape latency (s) during test days using the morris water maze (MWM) test following hypoandrogenic induction and chrysin treatment in study groups. Values are mean ± SD. p < 0.05 are significant,* p < 0.05 versus control, and p < 0.05 versus hypoandrogenic group.

Spatial learning in the tested rats was assessed using the MWM test. All groups were able to locate the platform within four days of training. The hypoandrogenic group spent significantly more time finding the platform compared to the control group. The hypoandrogenic + chrysin group at a dose of 75 mg required significantly less time to reach the platform than the hypoandrogenic group (p < 0.05). No significant difference in escape latency was observed between the hypoandrogenic + chrysin group at a dose of 50 mg and the hypoandrogenic group (Figure 3).

#### 3.2.2 Distance Traveled to Target Quadrant During Training Days and Test Day in MWM

**FIGURE 4:**
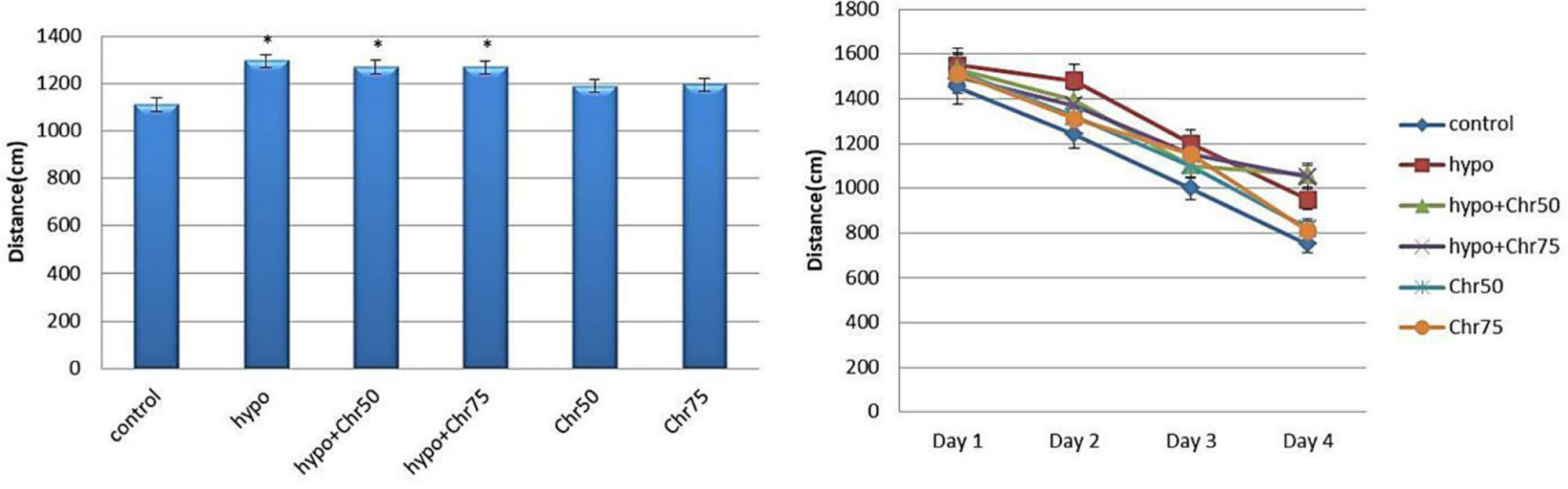
Evaluation of distance traveled (cm) in the target quadrant during test days using the morris water maze (MWM) test following hypoandrogenic induction and chrysin treatment in study groups. Values are mean ± SD. p < 0.05 are significant,* p < 0.05 versus control, and p < 0.05 versus hypoandrogenic group.

Data from the MWM test revealed that the hypoandrogenic group showed a significant increase in the average distance traveled within the target quadrant compared to the control group (p < 0.05). Treatment with chrysin at doses of 75 mg and 50 mg in the hypoandrogenic + chrysin groups led to decreased distance traveled compared to the hypogonadism group. However, this reduction was not statistically significant (Figure 4).

#### 3.2.3 Swimming Speed in MWM

**FIGURE 5:**
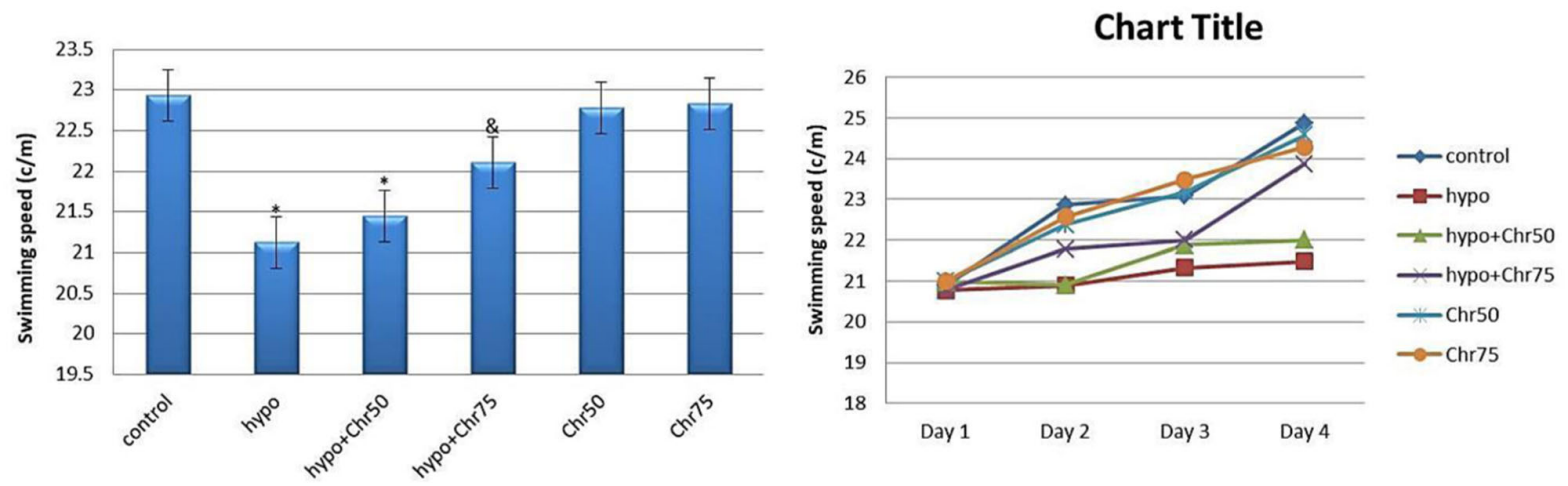
Evaluation of the average swimming speed (c/m) in the target quadrant during test days using the morris water maze (MWM) test following hypoandrogenic induction and chrysin treatment in study groups. Values are mean ± SD. p < 0.05 are significant,* p < 0.05 versus control, and p < 0.05 versus hypoandrogenic group.

Statistical analysis results indicated a noticeable decrease in swimming speed in the hypoandrogenic group and hypoandrogenic + chrysin group at a dose of 50 mg compared to the control group. Test day results showed that in the hypoandrogenic + chrysin group at a dose of 75 mg, swimming speed increased following hypoandrogenism compared to the hypoandrogenic group (p < 0.05). No significant difference in swimming speed was observed between the hypoandrogenic + chrysin group at a dose of 50 mg and the hypoandrogenic group (Figure 5).

#### 3.2.4 Time Spent in Target Quadrant in MWM

**FIGURE 6:**
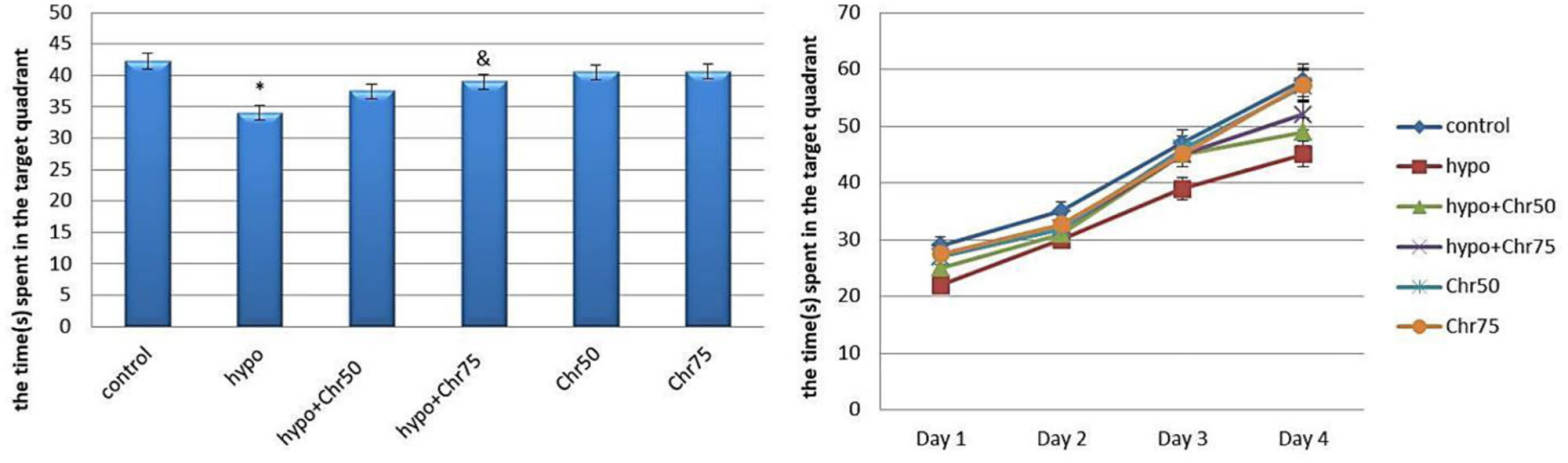
Evaluation of the average spent time(s) in the target quadrant during test days using the morris water maze (MWM) test following hypoandrogenic induction and chrysin treatment in study groups. Values are mean ± SD. p < 0.05 are significant,* p < 0.05 versus control, and p < 0.05 versus hypoandrogenic group.

Analysis of time spent in the target quadrant during the MWM test revealed that the hypoandrogenic group spent significantly less time in the target quadrant compared to both the hypoandrogenic + chrysin group at a dose of 75 mg and the control group (p < 0.05). Meanwhile, no significant difference was observed between the hypoandrogenic group and the hypoandrogenic + chrysin group at a dose of 50 mg (Figure 6).

Groups receiving chrysin alone at doses of 50 and 75 mg showed no significant difference in escape latency, distance traveled, swimming speed, and time spent in the target quadrant compared to the control group.

### 3.3 Real‐Time Polymerase Chain Reaction mRNA Analysis

#### 3.3.1 Analysis of Caspase‐3, BAX, and Bcl2 mRNA Expression

**FIGURE 7:**
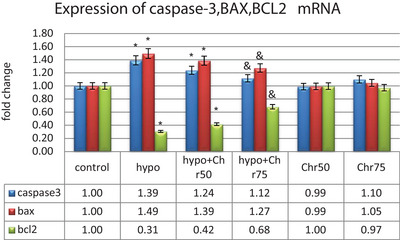
Evaluation of mRNA expression BAX, BCL2, and Caspase‐3 following hypoandrogenic induction and chrysin treatment in the hippocampus tissue as measured by the real‐time PCR. Values are mean ± SD. p < 0.05 are significant, * p< 0.05 versus control, and p < 0.05 versus hypoandrogenic group.

The expression of the pro‐apoptotic genes Caspase‐3 and BAX showed a significant increase in the hypoandrogenic groups compared to the control group in hippocampal tissue. Furthermore, the hypoandrogenic + chrysin group at a dose of 75 mg exhibited a significant decrease in the expression of these genes compared to the hypoandrogenic group (p ≤ 0.05). Analysis of Bcl2 gene expression, as an anti‐apoptotic gene, showed a significant decrease in its expression in the hypoandrogenic group compared to the control group. Additionally, the hypoandrogenic + chrysin group at a dose of 75 mg showed significant increases in Bcl2 gene expression compared to the hypoandrogenic group (p ≤ 0.05). Groups receiving chrysin alone at doses of 50 and 75 mg showed no significant difference in the expression levels of Caspase‐3, BAX, and Bcl2 mRNA compared to the control group (Figure 7).

#### 3.3.2 Expression Analysis of LC3β, Beclin‐1, and Atg5, 7, 12

**FIGURE 8:**
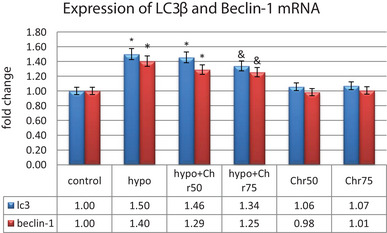
Evaluation of mRNA expression of Lc3β and Beclin‐1 following hypoandrogenic induction and chrysin treatment in the hippocampus tissue as measured by the real‐time PCR. Values are mean ± SD. p < 0.05 are significant, * p < 0.05 versus control, and p < 0.05 versus hypoandrogenic group.

**FIGURE 9:**
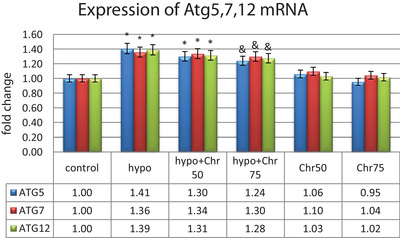
Evaluation of mRNA expression of ATG5, 7, 12 following hypoandrogenic induction, and chrysin treatment in the hippocampus tissue as measured by the real‐time PCR. Values are mean ± SD. p < 0.05 are significant, * p < 0.05 versus control, and p < 0.05 versus hypoandrogenic group.

Data from real‐time demonstrated that the expression of LC3β, Beclin‐1, and Atg5, 7, 12 genes, as autophagy markers, exhibited normal mRNA expression levels in the control group. Hypoandrogenic induction led to a significant increase in the expression of autophagy marker mRNAs compared to the control group in hippocampal tissue. Chrysin treatment in the hypoandrogenic + chrysin group at a dose of 75 mg significantly reduced the mRNA levels of LC3β, Beclin‐1, and Atg5, 7, 12 compared to the hypoandrogenic group (p ≤ 0.05). Chrysin treatment in the hypoandrogenic + chrysin group at a dose of 50 mg also decreased the expression of these genes, but this reduction was not statistically significant. Groups receiving chrysin alone at doses of 50 and 75 mg showed no significant difference in the expression levels of LC3β, Beclin‐1, and Atg5, 7, 12 mRNA compared to the control group (Figures 8 and 9).

#### 3.3.3 Expression Analysis of MicroRNAs: miR‐30a, miR‐375, and miR‐204

**FIGURE 10:**
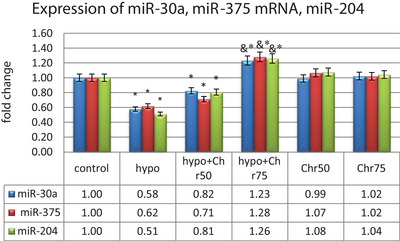
Evaluation of microRNA expression miR‐30a, miR‐375, and miR‐204 following hypoandrogenic induction and chrysin treatment in the hippocampus tissue as measured by the real‐time PCR. Values are mean ± SD. p < 0.05 are significant, * p < 0.05 versus control, and p < 0.05 versus hypoandrogenic group.

The real‐time analysis of miR‐30a, miR‐375, and miR‐204 expression showed a significant decrease in their expression in the hypoandrogenic group compared to the control group in hippocampal tissue. Furthermore, the hypoandrogenic + chrysin groups at doses of 50 and 75 mg exhibited an increase in the expression of these microRNAs compared to the hypoandrogenic group. However, this increase was only statistically significant in the hypoandrogenic + chrysin group at a dose of 75 mg (p ≤ 0.05). Groups receiving chrysin alone at doses of 50 and 75 mg showed no significant difference in the expression levels of these microRNAs compared to the control group (Figure 10).

### 3.4 Biochemical Findings

#### 3.4.1 Determination of Serum Levels of Oxidative Stress Enzymes: MDA, SOD, and GPX

**FIGURE 11:**
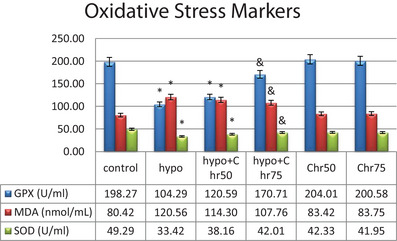
Evaluation of the oxidative stress markers (MDA, SOD, and GPX) following hypoandrogenic induction and chrysin treatment in in study groups. Values are mean ± SD. p < 0.05 are significant, * p < 0.05 versus control, and p < 0.05 versus hypoandrogenic group.

The biochemical analysis of the serum of the tested rats revealed a significant decrease in GPX and SOD enzyme levels in the hypoandrogenic group compared to the control group. A significant increase in serum MDA levels was also observed in the hypoandrogenic group compared to the control group. Chrysin treatment in both the hypoandrogenic + chrysin group at doses of 75 and 50 mg led to a relative improvement in the levels of these enzymes. However, only in the hypoandrogenic + chrysin group at a dose of 75 mg did we observe a significant decrease in MDA and a significant increase in GPX and SOD levels compared to the hypoandrogenic group (Figure 11) (p ≤ 0.05).

## 4 Discussion

The hippocampus is central to learning, memory, and stress responses, and its growth and function in adults largely depend on the pivotal role of androgens and their metabolites (Kight and McCarthy 2020). Changes in neurogenesis, memory, and learning have been associated with fluctuations in testosterone and estradiol levels in the bloodstream (Duarte‐Guterman et al. 2015). Observations have indicated that a reduction in androgens plays a significant role in impairing synaptic plasticity in the hippocampus, potentially laying the groundwork for neurological disorders in aging men or those with hypogonadism (Shehata 2010). In this study, we observed histological changes in the hippocampus and a decrease in its antioxidant defense mechanisms in the absence of testosterone, which likely leads to neurodegeneration in the hippocampus and ultimately impairs learning and memory acquisition in gonadectomized rats. However, some studies have shown that decreased levels of androgens or their receptors in the hippocampus are directly (rather than through oxidative stress induction or neuronal damage) associated with impairments in memory and learning (Beyenburg et al. 2000).

This study evaluated markers of cell death, including autophagy and apoptosis, as well as the density of dark neurons, following the induction of hypogonadism. Apoptosis is a programmed cell death mechanism, while autophagy is a natural intracellular degradation process; both play crucial roles in the functional growth of the nervous system (Chung et al. 2018). Normal autophagic activity is essential for enhancing the capacity of hippocampal neurons to form dendritic spines, increase synaptic strength, and ultimately form new memories (Li et al. 2014). However, abnormal autophagy can activate apoptotic cascades that ultimately lead to neuronal death (Yu et al. 2017). In the present study, increased autophagy markers (LC3II, Beclin‐1, Atg5, Atg7, and Atg12) in the hippocampus of gonadectomized rats were associated with elevated levels of BCL2, BAX, and caspase‐3 as apoptotic signals, indicating that apoptosis was induced due to excessive autophagy. Consistent with these changes, histological evaluations revealed a reduction in dark neuron density in the hippocampus, as well as impaired learning and memory in the MWM test in gonadectomized rats. Numerous studies have demonstrated the effects of androgen depletion on memory function and the induction of cell death processes (Choi et al. 2019; MacLusky et al. 2006; Muthu and Seppan 2020; Ramadan et al.). However, the specific signaling mechanisms by which androgens influence memory function or induce processes such as autophagy remain unclear.

To address this, this study evaluated the expression of regulatory microRNAs (miRNAs) miR‐30a, miR‐375, and miR‐204, which are associated with androgens or their receptors (Kraus et al. 2015; Lyu et al. 2017; Todorova et al. 2017). miRNAs are small non‐coding RNA molecules recognized as RNA silencers and post‐transcriptional regulators. MiR‐30a, miR‐375, and miR‐204 are expressed in the hippocampus (Khandelwal, Dey, Chakravarty, and Kumar 2019; H. Liu et al. 2021; X. Liu et al. 2015) and also regulate autophagy processes (Frankel and Lund 2012; Yang and Liang 2015). Studies have shown that miRNAs expressed in the hippocampus and cortex of adults may also be involved in synaptic plasticity and memory formation (Wang et al. 2012).The results of this study indicated that the expression of all three miRNAs was reduced in hypoandrogenic rats (the gonadectomized group), which aligned with a decrease in memory performance in these rats. Since miR‐30a, miR‐375, and miR‐204 are negative regulators of certain autophagy‐related genes, it was not unexpected to find an excessive increase in autophagy gene expression following a reduction in these miRNAs. In line with these findings, numerous studies have been conducted regarding the role of miR‐204, miR‐30a, and miR‐375 in regulating autophagy, hippocampal‐related diseases, and memory function (Khandelwal et al. 2019; H. Liu et al. 2021; X. Liu et al. 2015; Shan et al. 2017; Tao et al. 2021; Yang and Liang 2015).

Numerous studies have shown that disorders in the hippocampus leading to neuronal death and changes in memory and learning can be effectively addressed with antioxidant‐based therapies (Barichello et al. 2007; Hall et al. 2010). In this study, we investigated the protective effects of chrysin flavonoid on memory decline and neuronal death in the hippocampus affected by hypogonadism. Chrysin (5.7‐dihydroxyflavone) is one of the most active flavonoids found in nature, recognized for its antioxidant, anti‐apoptotic, and anti‐inflammatory properties used in the treatment of liver diseases, neurodegenerative disorders, and reproductive system conditions (Eldutar, Kandemir, Kucukler, and Caglayan 2017). It has been demonstrated that chrysin exerts its neuroprotective effects primarily by reducing reactive oxygen species (ROS) and lipid peroxidation (Nabavi et al. 2015). The present study showed that chrysin (75 mg) improved the levels of antioxidant enzymes MDA, SOD, and GPX following gonadectomy in rats. Additionally, it was associated with increased expression of anti‐apoptotic proteins BCL2 and decreased expression of pro‐apoptotic proteins BAX and caspase‐3 in hippocampal tissue. Furthermore, chrysin administration reduced the excessive expression of autophagy marker genes in gonadectomized rats. In this context, some studies demonstrated that chrysin mitigated apoptosis induced by excessive autophagy in the hippocampus of sleep‐deprived mice (Cao et al. 2019). An increasing number of emerging studies have highlighted the effects of chrysin in various neurological disorders, including Alzheimer's disease, Parkinson's disease, epilepsy, multiple sclerosis, ischemic stroke, traumatic brain injuries, and brain tumors, noting improvements in motor and cognitive impairments as among its capabilities (Mishra et al. 2021). In our study, data from the MWM experiment indicated that treatment with chrysin (75 mg dose) improved memory impairment due to gonadectomy. According to the molecular data from this study, treatment with chrysin in gonadectomized rats effectively normalized the expression levels of miR‐30a, miR‐375, and miR‐204. Recent studies have suggested that apart from their antioxidant potential, flavonoids play a crucial role in modulating miRNAs related to diseases such as cancer. Flavonoids exert their effects on targets through direct binding to miRNAs or by interacting with relevant proteins associated with miRNAs.

## 5 Conclusion

In the present study, chrysin flavonoid led to a reduction in autophagy and improved memory function in the hippocampal tissues of hypogonadal rats, which may occur either directly through the regulation of antioxidant enzyme levels or indirectly by modulating the expression of miR‐30a, miR‐375, and miR‐204.

## Author Contributions

**Amir‐Hossein Ebadi**: investigation, data curation. **Maryam Moghimian**: writing – review and editing, project administration. **Seyd‐Hossein Abtahi‐evari**: methodology, software. **Zahra Saadatian**: methodology. **Alireza Fathi**: conceptualisation. **Malihe Soltani**: writing – original draft.

## Ethics Statement

This study received approval from the Ethics Committee of the Ministry of Health, Treatment, and Medical Education in the Islamic Republic of Iran with the approval number: IR.GMU.REC.1401.089, dated November 9, 2023.

### Peer Review

The peer review history for this article is available at https://publons.com/publon/10.1002/brb3.70529.

## Data Availability

The data that support the findings of this study are available from the corresponding author upon reasonable request.
